# Inhibition of retroviral Gag assembly by non-silencing miRNAs promotes autophagic viral degradation

**DOI:** 10.1007/s13238-017-0461-z

**Published:** 2017-09-07

**Authors:** Na Qu, Zhao Ma, Mengrao Zhang, Muaz N. Rushdi, Christopher J. Krueger, Antony K. Chen

**Affiliations:** 1 Department of Biomedical Engineering, College of Engineering, Peking University, Beijing, 100871 China; 2 Wallace H Coulter Department of Biomedical Engineering, Georgia Institute of Technology, Atlanta, GA 30332 USA

**Keywords:** microRNA, Gag protein, autophagy

## Abstract

We recently reported an unconventional mechanism by which miRNAs inhibit HIV-1 viral production. This occurs when miRNAs bind nonspecifically to the viral structural protein Gag, interfering with viral RNA-mediated Gag assembly at the plasma membrane. Consequently, misassembled viral complexes are redirected into the endocytic pathway where they are delivered to lysosomes for degradation. In this study, we demonstrate that autophagy is a critical mediator of the viral degradation pathway and that this pathway is not HIV-1 specific. Misassembled viral complexes were found to colocalize extensively with LC3 and p62 in late endosomes/lysosomes, demonstrating a convergence of autophagy with functional degradative compartments. Knocking down autophagosome formation machineries reduced this convergence, while treatment with autophagy-inducer rapamycin enhanced the convergence. Furthermore, similar autophagy-dependent nonspecific miRNA inhibition of murine leukemia virus (MLV) assembly was shown. Overall, these results reveal autophagy as a crucial regulator of the retroviral degradation pathway in host cells initiated by nonspecific miRNA-Gag interactions. These findings could have significant implications for understanding how cells may regulate retroviral complex assembly by miRNA expression and autophagy, and raise the possibility that similar regulations can occur in other biological contexts.

**Electronic supplementary material:**

The online version of this article (doi:10.1007/s13238-017-0461-z) contains supplementary material, which is available to authorized users.

## Introduction

MicroRNAs (miRNAs) are small noncoding RNAs, 19–24 nucleotides in length, with gene-silencing functions critical to the regulation of numerous physiological and pathological processes (Filipowicz et al., 2008; Sharp, 2009). To achieve gene silencing, miRNAs associate with the RNA-induced silencing complex (RISC) to target specific mRNAs for degradation or translational repression. In addition to this role, emerging evidence has shown that miRNAs can also interact with other proteins, impacting cellular physiology via a variety of mechanisms independent of gene silencing (Chen et al., 2014; Eiring et al., 2010; Fabbri et al., 2012; Lehmann et al., 2012; Prud’homme et al., 2016; Ranganathan et al., 2017; Vickers et al., 2011; Yelamanchili et al., 2015). We previously discovered one such unconventional miRNA function, wherein miRNA acts as a potent inhibitor of the formation of HIV-1 virus particles (Chen et al., 2014).

Gag is the main retroviral structural protein that orchestrates the formation of HIV-1 virus particles. To drive viral assembly, thousands of Gag molecules must coalesce around the viral genome to form a highly-ordered Gag multimer at the plasma membrane (PM) (Briggs et al., 2004). To date, much evidence has shown that the process is strongly dependent on interaction between the nucleocapsid domain of Gag (NC) and the viral genome. One major interaction involves specific binding of NC to the psi-element in the viral genome, enabling its selective recruitment to the PM from the complex cytoplasmic environment (Aldovini and Young, 1990; Berkowitz et al., 1993; Gorelick et al., 1990). Another interaction involves nonspecific binding of NC to regions outside the psi-element, which is thought to facilitate Gag coalescence and multimerization at the PM (Kutluay and Bieniasz, 2010; Kutluay et al., 2014; Muriaux et al., 2001; Rulli et al., 2007). Recently, we showed that miRNAs can also bind to NC, forming miRNA-Gag complexes that block viral formation by disrupting viral RNA-mediated Gag assembly at the PM (Chen et al., 2014). This effect was more pronounced when the miRNAs did not participate in RNAi (nonsense miRNAs with no complementary target sites in endogenous mRNAs or the viral genome). The resulting misassembled viral complexes were then endocytosed and ultimately delivered to lysosomes for degradation. However, the question of why the misassembled viral complexes were routed to lysosomes, as opposed to other destinations, following endocytosis remains to be clarified.

Autophagy is a naturally-occurring catabolic process in which cellular homeostasis and survival are maintained through the removal and recycling of unwanted cellular materials in lysosomes (Klionsky, 2007; Mizushima, 2007). There are three main types of autophagy: macroautophagy, microautophagy, and chaperone-mediated autophagy. Of these, macroautophagy is the most primitive and well-studied form of autophagy (Feng et al., 2014). During this process, target substrates are enclosed within cytosolic double-membrane delivery vesicles termed autophagosomes. Subsequent fusion of autophagosomes with late endosomes and/or lysosomes enables the contents to be decomposed into macromolecular constituents that can be recycled. Macroautophagy has been observed in the clearance of surplus and damaged organelles (Liu and Czaja, 2013; Rambold and Lippincott-Schwartz, 2011), proteins and ribonucleoprotein aggregates (Frankel et al., 2017; Wong et al., 2012), and pathogens (Mizushima et al., 2008). These unique functions of macroautophagy thus prompted us to investigate its potential role in the miRNA-mediated viral degradation pathway. Here, we provide evidence for a pivotal role of macroautophagy in regulating the delivery of misassembled viral complexes to lysosomes. Additionally, we demonstrate a similar regulation by miRNAs and role of autophagy in disrupting the formation of murine leukemia virus (MLV), a retrovirus that belongs to a different genus from HIV-1. These findings linking miRNAs and autophagy could facilitate the understanding of how cellular virus resistance could be enhanced, benefiting the design of generalizable therapeutic approaches through miRNA expression or autophagy-induction.

## Results and Discussion

### Non-silencing miRNAs mediate the redirection of HIV-1 Gag complexes from the plasma membrane to lysosomes

**Figure 1:**
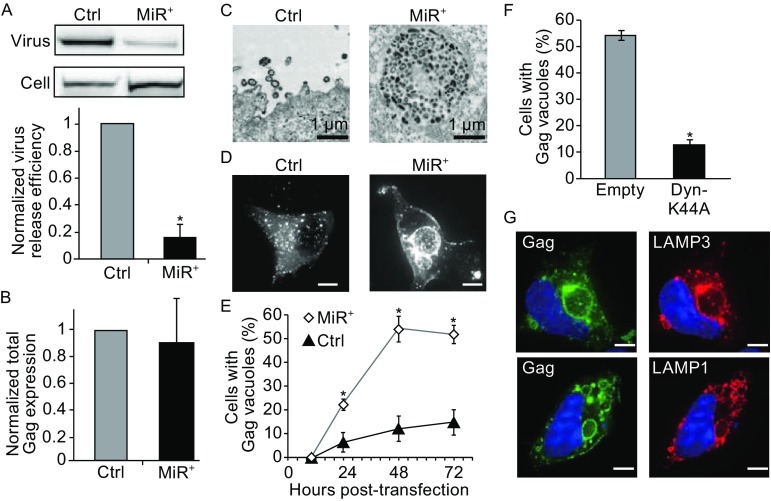
**Overexpression of non-silencing miRNAs causes redistribution of HIV-1 Gag complexes from the PM to lysosomes through endocytosis**. (A) The effect of overexpressing non-silencing miRNAs on virus particle release in MiR^+^ and Ctrl cells at 48 h post-transfection of pNL43ΔPΔE. Western blot was performed with pooled Ig from HIV-1-infected patients (HIV-Ig) to detect Gag in viral particles and cells. Virus release efficiency was calculated as described in MATERIALS AND METHODS, normalized to virus release efficiency in Ctrl cells. (B) Total Gag expression levels (virus Gag plus cellular Gag) measured by Western blot in MiR^+^ and Ctrl cells after transfection with pNL43ΔPΔE, normalized to total Gag expression in Ctrl cells. (C) Representative electron micrograph images of MiR^+^ and Ctrl cells transfected with pNL43ΔPΔE. Characteristic Gag-enriched viral buds are observed at the PM of Ctrl cells, while MiR^+^ cells tend to form large intracellular vacuoles. (D) Representative fluorescence images of Gag expression in MiR^+^ and Ctrl cells through pNL43ΔPΔE-Gag-EGFP transfection. (E) Post-transfection time-course quantification of the percentage of cells with intracellular Gag-enriched vacuoles with diameter greater than 1 μm. (F) Co-transfection of pNL43ΔPΔE-Gag-EGFP with Dyn-K44A, a dominant-negative mutant that inhibits endocytosis, but not with empty vector, led to significant reduction in Gag vacuole formation. (G) Representative images of LAMP3 and LAMP1 in MiR^+^ cells expressing pNL43ΔPΔE-Gag-EGFP. LAMP3 and LAMP1 colocalize extensively with Gag-EGFP in large vacuoles. Data represent mean ± SD of at least three replicates. Each replicate experiment was performed by examining at least 200 transfected cells. Unless otherwise noted, scale bar = 10 μm

To demonstrate the ability of non-silencing miRNAs to alter intracellular redistribution of HIV-1 Gag complexes, we prepared HEK 293 cell lines stably expressing either the exogenous miRNA hsa-miR-146a (MiR^+^) or an empty vector (Ctrl), and transfected both with pNL43ΔPΔE, an HIV-1 proviral clone construct (See MATERIALS AND METHODS). Since hsa-miR-146a has no known target sites in the viral genome or the endogenous mRNAs of HEK 293 cells, we hypothesized that this non-silencing miRNA could function to disrupt viral assembly through mechanisms other than gene silencing. Supporting this possibility, HIV-1 Gag transfected MiR^+^ cells exhibited reduced capacity to form viral particles compared to Ctrl cells (Fig. 1A), assayed by calculating the amount of Gag in the supernatant relative to total Gag in cells and the supernatant. Additionally, total Gag expression was similar in MiR^+^ and in Ctrl cells (Fig. 1B), suggesting that hsa-miR-146a could not cause silencing of viral genes in HEK 293 cells. Thus, we concluded that the observed reduction in virus release in MiR^+^ cells was not caused by miRNA-mediated gene silencing.

The reduction in virus release, despite the similar total level of Gag expression in MiR^+^ and Ctrl cells, raised the possibility that non-silencing miRNAs function to alter intracellular distribution of Gag complexes. Supporting this idea, electron micrographs and fluorescence microscopy both showed that HIV-1 Gag localized to large vacuoles within MiR^+^ cells while appear to cluster at the PM and exhibit typical viral budding characteristics in Ctrl cells (Fig. 1C and 1D). Large Gag vacuoles, identified as Gag-containing vacuoles with diameters greater than 1 μm, were present in nearly 50% of MiR^+^ cells but only in ~10% of Ctrl cells assessed at 48 h post-transfection of the viral constructs (Fig. 1E). The increased vacuole formation was driven in part by clathrin-mediated endocytosis, as inhibiting the endocytosis machinery using dominant-negative dynamin-K44A (Dyn-K44A) significantly reduced vacuole formation (Fig. 1F). Furthermore, immunofluorescence imaging with lysosome-associated membrane protein (LAMP)-specific antibodies identified the Gag-containing vacuoles as late endosomes and lysosomes (Fig. 1G). Similar results were obtained when analogous experiments were performed in a second cell line that stably expresses hsa-miR-888 (MiR^+^888), a non-silencing miRNA that has no sequence homology with miR-146a (Figs. S1 and S2). Thus, under conditions where cells express non-silencing miRNAs, misassembled viral complexes are targeted from the PM to lysosomes via endocytosis.

**Figure 2:**
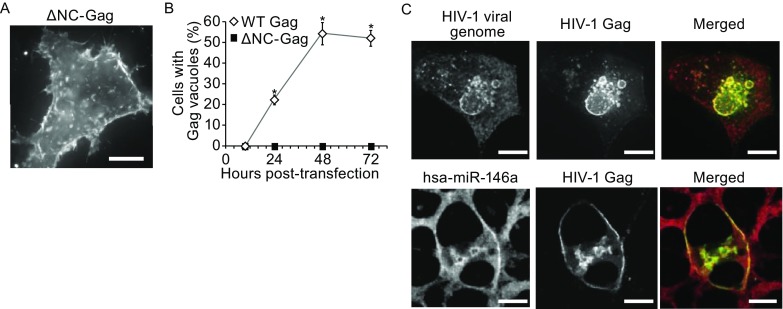
**Interaction of miRNAs with HIV-1 Gag proteins drives intracellular Gag redistribution**. (A) Representative fluorescence micrograph of ΔNC-Gag in MiR^+^ cells. MiR^+^ cells were transfected with pNL43ΔPΔEΔNC-Gag-EGFP and imaged at 48 h post-transfection. Note that ΔNC-Gag does not form intracellular vacuoles. (B) Time-course quantification of Gag vacuole formation by ΔNC-Gag as compared with wildtype Gag in MiR^+^ cells. Data represent mean ± SD of at least three replicates. Each replicate experiment was performed by examining at least 200 transfected cells. (C) Representative images of hsa-miR-146a or HIV-1 viral genome (detected by FISH) and Gag (detected by Gag-EGFP) in MiR^+^ cells transfected with pNL43ΔPΔE-Gag-EGFP. Scale bar = 10 μm

Large vacuoles were not observed in MiR^+^ cells when Gag was not expressed (Fig. S3). This suggests that vacuole formation results from interaction between the non-silencing miRNA and Gag. To test this possibility, we questioned if vacuoles could still form in cells expressing mutant Gag lacking NC (ΔNC-Gag). Results indicated that large Gag vacuoles could not form in MiR^+^ or Ctrl cells (Fig. 2A and 2B), suggesting that miRNA-NC interactions are necessary for large Gag vacuole formation. Supporting this, fluorescence *in situ* hybridization (FISH) experiments showed that non-silencing miRNAs and HIV-1 viral RNAs could colocalize with Gag at the PM and in vacuoles in MiR^+^ cells (Fig. 2C). Thus, together with the finding that Gag exhibited reduced capacity to form viral particles, these results confirm our previous finding that miRNAs and Gag can interact to disrupt viral assembly at the PM, with the misassembled viral complexes being sequestered in lysosomes following endocytosis.

### Autophagy interacts with the endocytosis-mediated lysosomal degradation pathway to drive degradation of misassembled viral complexes

Following endocytosis, the observation that misassembled viral complexes are sequestered to lysosomes, as opposed to transiting to other destinations such as the trans-Golgi network or the extracellular milieu through exocytosis suggests that lysosomal delivery of misassembled viral complexes is a highly-regulated process. One possible regulation is through macroautophagy, which deliver unwanted materials to lysosomes for degradation. Increasing evidence suggests that macroautophagy could intersect with the endocytosis-mediated lysosomal degradation pathway (Sanchez-Wandelmer and Reggiori, 2013). Given that the misassembled Gag complexes appear to be highly sequestered in the late endosomes of MiR^+^ cells, we sought to investigate whether this sequestration is autophagy-dependent.

**Figure 3:**
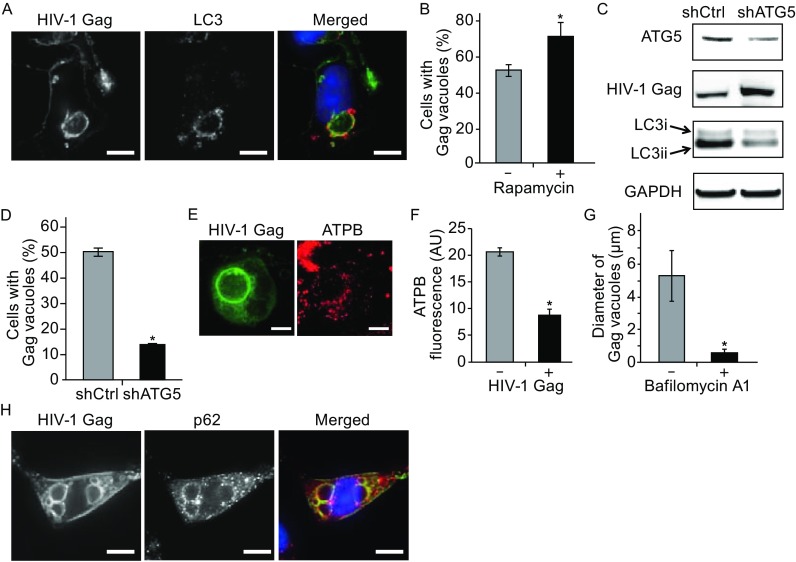
**The role of autophagy in mediating lysosomal delivery of misassembled viral complexes**. (A) Representative images of LC3 and HIV-1 Gag in MiR^+^ cells. LC3 and HIV-1 Gag colocalize at the vacuoles. (B) Increase in Gag vacuole formation in response to rapamycin treatment. 24 h after MiR^+^ cells were transfected with pNL43ΔPΔE-Gag-EGFP constructs, rapamycin (200 nmol/L) or DMSO control were added. Percentage of Gag-expressing MiR^+^ cells containing at least one vacuole were assayed by fluorescence microscopy at 24 h following treatment. (C) The effect of ATG5 knockdown on HIV-1 Gag and LC3 conversion using shRNA against ATG5 (shATG5). At 48 h post-transfection, Western blot was performed to assess knockdown efficiency and its effect on Gag and LC3 conversion. shATG5 reduced the level of ATG5 by ~40%. This reduction was accompanied by ~50% reduction in LC3 conversion (calculated as the ratio of LC3ii to GAPDH) and a nearly two-fold increase in HIV-1 Gag accumulation in cells compared with cells transfected with the shCtrl plasmid. (D) Knockdown of ATG5 led to a reduction in Gag vacuole formation, assayed by fluorescence microscopy. (E) MiR^+^ cells expressing pNL43ΔPΔE-Gag-EGFP were fixed and immunofluorescently-labeled with anti-ATPB antibodies to stain mitochondria. (F) Quantification of total mitochondrial ATPB signal per cell. (G) Decrease in Gag vacuole diameter in response to bafilomycin A1 treatment. 24 h after MiR^+^ cells were transfected with pNL43ΔPΔE-Gag-EGFP constructs, 50 nmol/L bafilomycin A1 or DMSO control were added. Following 18 h incubation, cells were imaged for the presence of vacuoles. (H) Representative images of p62 and HIV-1 Gag in MiR^+^ cells. p62 and HIV-1 Gag colocalize at the vacuoles. For (B) and (D), data represent mean ± SD of three replicates, with each replicative experiment performed by examining at least 200 transfected cells. For (F), data represent mean fluorescence intensity ± SD of 50 cells either untransfected or transfected with pNL43ΔPΔE-Gag-EGFP. For (G), data represent mean diameter ± SD of at least 200 individual vacuoles. Scale bar = 10 μm

To test this, we characterized autophagy activity in MiR^+^ and Ctrl cells. Western blot analysis of LC3, an indicator of autophagy initiation and a marker of autophagosomes, showed that total LC3 levels and LC3 conversion capacity in MiR^+^ and in Ctrl cells are similar (Fig. S4). This suggests overexpressing non-silencing miRNAs does not impact LC3 gene expression or conversion capacity. However, immunofluorescence with antibodies against LC3 showed different intracellular LC3 distribution patterns in Gag-expressing MiR^+^ cells as compared with Ctrl cells (Figs. 3A and S5). In Ctrl cells, LC3 and HIV-1 Gag proteins both exhibited a dispersedly-distributed and punctate staining pattern in the cytosol but did not colocalize with each other. In MiR^+^ cells, LC3 localized extensively at the periphery and in the lumen of the Gag-enriched late endosomes/lysosomes. Thus, it appears that under conditions where misassembled viral complexes are internalized via endocytosis, autophagosomes are redistributed to the endocytosed complexes and participate in virus degradation.

To further investigate the dependence on autophagy in directing the endocytosed complexes to lysosomes for degradation, we tested if altering autophagic activity through pharmacological treatment or shRNA knockdown could impact the formation of intracellular vacuoles enriched in Gag, LC3, and LAMP proteins. Treatment with rapamycin, an autophagy enhancer, led to an increase in vacuole formation (Fig. 3B). Conversely, knockdown of ATG5, part of the machinery which drives autophagosome formation, caused a significant reduction in LC3 conversion, in the number of the Gag-enriched vacuoles, and an overall increase in Gag levels in MiR^+^ cells (Fig. 3C and 3D), as expected if inhibiting autophagy activity reduces viral degradation. Furthermore, knockdown of ULK1, a key regulator of autophagy induction and progression, also led to a reduction in vacuole formation, confirming large Gag vacuoles formed through autophagosome-mediated pathways (Fig. S6). Additional evidence showing that macroautophagy was indeed functional under conditions where misassembled viral complexes are delivered to lysosomes was obtained from immunofluorescence labeling experiments that showed sequestration and degradation of mitochondria in LC3-containing Gag vacuoles. Specifically, in cells containing large Gag vacuoles, mitochondria, immunostained with antibodies against mitochondrial ATP synthase submit unit beta (ATPB), appeared highly fragmented, with the total signal intensity significantly reduced as compared with that of cells not expressing the viral constructs (Fig. 3E and 3F). Additionally, mitochondria were observed within the large Gag vacuoles, and treatment with leupeptin to inhibit protease activity led to ATPB accumulation and increased Gag enrichment inside the lumen, not just at the vacuole periphery (Fig. S7), suggesting that the large vacuoles originated from macroautophagy and mediate rapid lysosomal proteolytic degradation of both cellular and viral substrates.

Treatment with bafilomycin A1, an inhibitor of vacuolar H^+^-ATPase that blocks autophagosomal/autolysosomal acidification in cells, reduced the size of Gag vacuoles significantly as compared with DMSO-treated cells (Fig. 3G). This supports the idea that large Gag vacuole formation is driven by fusion of autophagosomes with late endosomes/lysosomes. Interestingly, large Gag vacuoles were labeled by immunostaining with antibodies against p62 (Fig. 3H), which interacts with LC3 and mediates autophagic clearance of protein aggregates (aggrephagy). Thus, misassembled viral complexes could potentially resemble aggregates of malformed proteins, which trigger their degradation through an aggrephagy-mediated pathway. Altogether, these findings indicate that under conditions where retroviral assembly at the PM is disrupted by miRNAs resulting in endocytosis of viral complexes, macroautophagy plays a pivotal role in directing the misassembled complexes to lysosomes for degradation. The observed direction of misassembled viral complexes to lysosomes through autophagy, as opposed to other possible routes, could potentially serve as an innate defense mechanism by which cells utilize non-silencing miRNAs to disrupt HIV-1 infection and dissemination.

### Non-silencing miRNAs disrupt VLP formation and alter intracellular localization of MLV Gag and HIV-1 Gag through similar mechanisms

The ability of miRNAs to disrupt HIV-1 viral particle formation and cause viral degradation through autophagy led us to examine whether other retroviruses are regulated via similar mechanisms. Among retroviruses, MLV closely resembles HIV-1 in both assembly and budding, but belongs to a distinct viral genus (Jin et al., 2009; Jouvenet et al., 2006; Kutluay et al., 2014; Muriaux et al., 2004; Muriaux et al., 2001; Ono et al., 2005; Rein et al., 1994; Rulli et al., 2007). For example, Gag proteins of both viruses possess a nucleocapsid domain (NC) that is capable of interacting with RNAs nonspecifically (Kutluay et al., 2014; Muriaux et al., 2004; Rein et al., 1994). In addition, besides the viral genome, long-stranded cellular mRNAs can serve as scaffolds to facilitate Gag assembly, leading to formation of virus-like particles (VLPs) (Muriaux et al., 2001; Rulli et al., 2007). Furthermore, the PM is the major site of viral assembly in most cell types (Jin et al., 2009; Jouvenet et al., 2006; Ono et al., 2005). Therefore, since miRNAs can block formation of HIV-1 viral particles by interfering with Gag multimerization at the PM through interaction with NC and elicit autophagy-dependent viral degradation, we hypothesized that VLP formation and intracellular fate of MLV Gag complexes exhibit similar miRNA- and autophagy-mediated regulation. To test this possibility, MiR^+^ and Ctrl cells were transfected with CMV-driven plasmid constructs encoding MLV Gag (See MATERIALS AND METHODS). For comparison, analogous experiments were performed with plasmids encoding CMV-driven HIV-1 Gag or simian foamy virus (SFV) Gag (See MATERIALS AND METHODS). The latter experiment serves as a negative control because SFV budding, unlike HIV-1 or MLV budding, requires envelope protein (Linial, 1999; Mullers, 2013; Shaw et al., 2003), and therefore should not occur in these experimental conditions.

**Figure 4:**
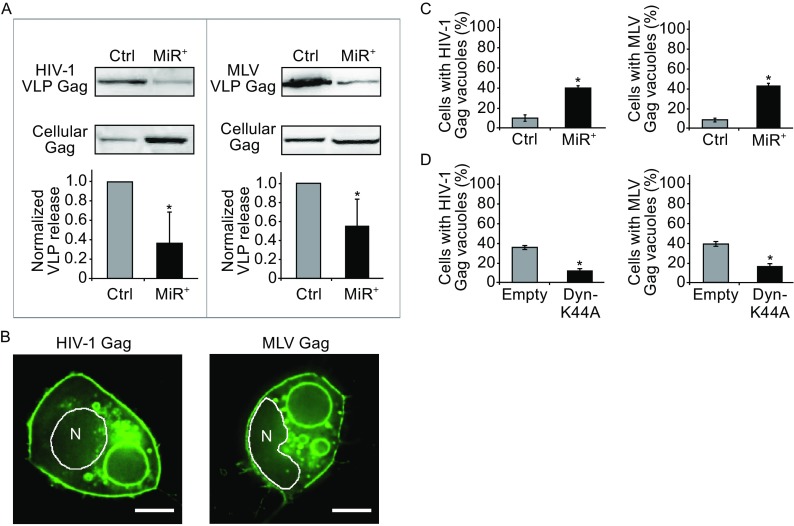
**The effects of overexpressing non-silencing miRNAs on Gag assembly and intracellular localization of various retroviruses**. (A) The effect of overexpressing non-silencing miRNAs on VLP production by MiR^+^ and Ctrl cells transfected with CMV-driven plasmids encoding HIV-1 or MLV Gag proteins. Results were normalized to virus release efficiency in Ctrl cells. (B) Representative images of HIV-1 or MLV Gag (detected by Gag-EGFP) in MiR^+^ cells. (C) Percentage of cells with large (diameter > 1 μm) intracellular vacuoles enriched in Gag after transfection of HIV-1 or MLV Gag. (D) Cells expressing HIV-1 or MLV Gag along with Dyn-K44A, a dominant-negative mutant that inhibits endocytosis, showed reduced Gag vacuole formation as compared with cells expressing an empty vector. Data represent mean ± SD of at least three replicates. For (C) and (D), each replicate experiment was performed by examining at least 200 transfected cells. Scale bar = 10 μm

VLP release efficiency, assayed at 48 h post-transfection of the CMV-driven constructs, showed that, similar to HIV-1 Gag, MLV Gag exhibited a reduced capacity to form VLPs in MiR^+^ cells as compared with Ctrl cells (Fig. 4A). In contrast, no VLP release was detected in MiR^+^ or Ctrl cells transfected with SFV Gag, as expected since envelope protein was not expressed (Fig. S8). Consistent with these findings, fluorescence microscopy imaging showed that both HIV-1 Gag and MLV Gag exhibited reduced puncta formation and an increased localization in large vacuoles in MiR^+^ cells as compared with Ctrl cells (Fig. 4B and 4C). In contrast, no difference in the localization of SFV Gag was observed in MiR^+^ or Ctrl cells. In both cells, SFV Gag formed intracellular aggregates, reflecting the inability to undergo viral budding at the PM in the absence of envelope protein (Fig. S9). Similar results were also obtained in MiR^+^888 cells as compared with Ctrl cells (Figs. S10 and S11). Thus, it appears that over-expressing non-silencing miRNAs can disrupt MLV Gag assembly into VLPs at the PM and lead to internalization of the resulting misassembled Gag complexes. Supporting this possibility, co-expression of MLV Gag with Dyn-K44A significantly reduced large Gag vacuole formation (Figs. 4D and S12), which was also observed in cells expressing both HIV-1 Gag and Dyn-K44A. This suggests that the observed intracellular accumulation of MLV or HIV-1 Gag was largely due to endocytosis of Gag complexes at the PM.

**Figure 5:**
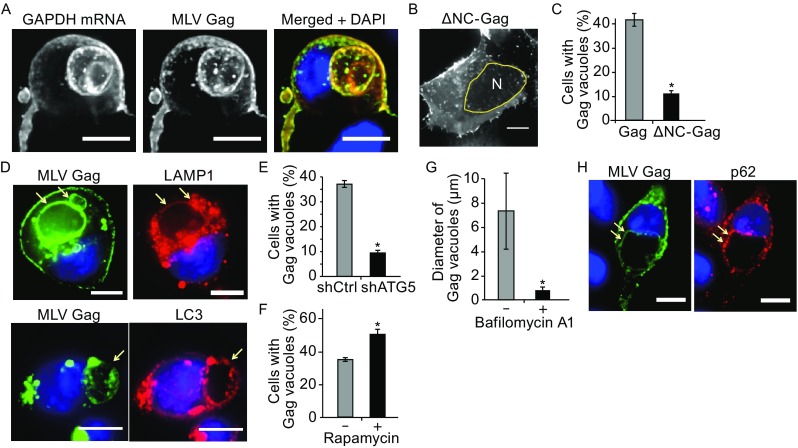
**The effect of RNA-Gag interactions and autophagy on lysosomal delivery of misassembled MLV Gag complexes**. (A) Representative images of GAPDH mRNA, detected by FISH, and Gag, detected by Gag-EGFP, in MiR^+^ cells transfected with MLV Gag-EGFP. (B) Representative image of MLV-ΔNC-Gag, detected by MLV ΔNC-Gag-EGFP in MiR^+^ cells. N indicates nucleus. (C) Percentage of MiR^+^ cells with large (diameter > 1 μm) intracellular vacuoles at 48 h post-transfection of MLV Gag or MLV ΔNC-Gag. (D) Representative images of LC3 and LAMP1, detected by immunofluorescence, in MiR^+^ cells expressing MLV Gag-EGFP. Arrows point to the vacuoles. (E) Knockdown of ATG5 led to a reduction in Gag vacuole formation. (F) Treatment with rapamycin (200 nmol/L) led to increased Gag vacuole formation. (G) Decrease in Gag vacuole diameter in response to bafilomycin A1 treatment. (H) Representative images of p62 and MLV Gag in MiR^+^ cells. p62 and MLV Gag colocalize at the vacuoles. Arrows point to the vacuoles. For (C), (E), and (F), data represent mean ± SD of three replicates, with each replicative experiment performed by visually examining at least 200 transfected cells. For (G), data represent mean diameter ± SD of at least 200 individual vacuoles. Scale bar = 10 μm

To further examine the similarity between the effects of non-silencing miRNAs on MLV and HIV-1 Gag, we performed experiments to characterize the nature of the MLV Gag vacuoles. FISH experiments showed GAPDH mRNA, an abundant endogenous mRNA, colocalized with Gag at the PM and at the periphery of the Gag-enriched vacuoles (Fig. 5A). These results support the idea that Gag can use cellular mRNAs as assembly scaffolds and suggest that miRNAs can disrupt this process to facilitate internalization of misassembled Gag complexes through endocytosis. Supporting this idea, expression of MLV Gag lacking NC did not lead to the formation of large vacuoles in MiR^+^ cells (Fig. 5B and 5C), indicating RNA-Gag interaction is essential for the miRNA-mediated internalization of MLV Gag, as in HIV-1 Gag. Thus, these findings suggest that MLV Gag complexes, like those of HIV-1, undergo redistribution from the PM to cytoplasm when miRNAs compete with scaffolding RNAs for Gag binding. Furthermore, immunofluorescence experiments identified the vacuoles as autophagosomes and lysosomes (Fig. 5D), suggesting that macroautophagy converges with components of the endocytosis-mediated lysosomal degradation pathway. Lysosomal sequestration of MLV Gag was highly dependent on autophagy activity, as shRNA knockdown of ATG5 or ULK1 led to a significant reduction in large Gag vacuole formation, whereas treatment with rapamycin enhanced vacuole formation (Figs. 5E, 5F, and S13). Additionally, treatment with autophagy inhibitor bafilomycin A1 led to a reduction in the size of MLV Gag vacuoles (Fig. 5G), suggesting their formation requires maturation of autophagosomes and subsequent fusion with lysosomes. Finally, large Gag vacuoles are p62-positive, suggesting they result from aggrephagy-mediated degradation (Fig. 5H). Altogether, these findings suggest that non-silencing miRNAs can mediate the redistribution and clearance of retroviruses that rely on long-stranded RNAs as scaffolds for assembly and particle formation.

## Conclusion

MicroRNAs are predominantly regarded as gene silencers involved in the regulation of various cellular processes, including embryogenesis, cellular differentiation, and pathogenesis. In these contexts, miRNAs, associated with proteins of the RISC, prevent the expression of specific genes through hybridization with target mRNAs. Alternatively, increasing evidence has shown that miRNAs can also interact with proteins outside the RISC and that these interactions can influence cellular processes through mechanisms independent of gene silencing (Chen et al., 2014; Eiring et al., 2010; Fabbri et al., 2012; Lehmann et al., 2012; Prud’homme et al., 2016; Ranganathan et al., 2017; Vickers et al., 2011; Yelamanchili et al., 2015). In one such study, we showed that both exogenous and endogenous miRNAs, particularly those not involved in mediating gene silencing, can compete with viral RNA for HIV-1 Gag binding, forming miRNA-Gag complexes that prevent viral RNA-mediated Gag assembly into high-order multimers essential for viral budding. The misassembled viral complexes were ultimately redirected into the endocytic pathway where they were delivered to lysosomes for degradation (Chen et al., 2014).

**Figure 6:**
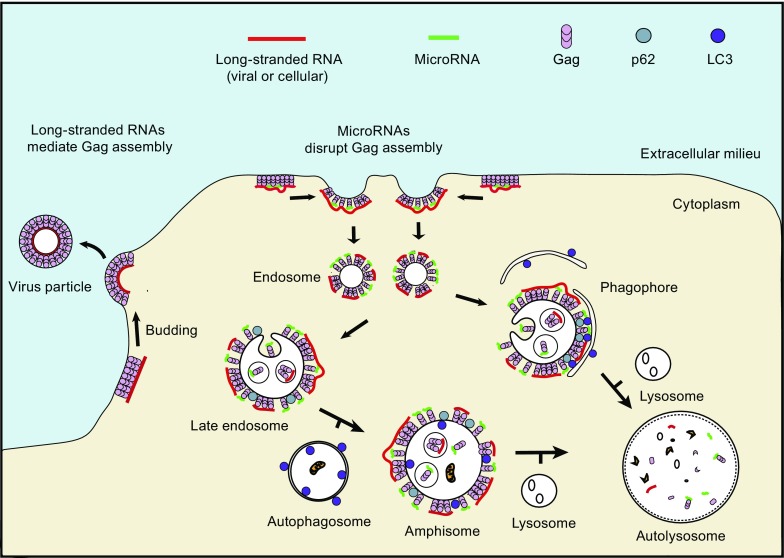
**Schematic model of how microRNAs and macroautophagy function to block virus production**. In the absence of Gag-miRNA complexes, Gag and scaffold RNAs (viral genome or long-stranded mRNAs) form stable complexes at the PM, resulting in viral budding. When Gag-miRNA complexes are present within the assembling complexes, particle formation is disrupted, resulting in internalization of misassembled complexes through endocytosis. The endocytosed Gag-miRNA complexes could be engulfed by phagophores, which mediates their autophagic degradation through a p62-dependent process. Additionally, fusion of autophagosomes to the late endosomes could form amphisomes, which can then mature into autolysosomes that degrade the enclosed misassembled viral complexes

In this study, we demonstrated that targeting of misassembled viral complexes from the PM to lysosomes for degradation is driven by macroautophagy (Fig. 6). The process involves p62, which potentially recognizes the endocytosed misassembled viral complexes as protein aggregates. Additionally, we showed that the assembly of MLV can also be regulated by miRNAs and autophagy in cells. The analogous effects of non-silencing miRNAs on the misassembly and degradation of HIV-1 and MLV Gag proteins suggest that the ability of Gag to interact nonspecifically with a diverse class of RNAs, as demonstrated previously (Campbell and Vogt, 1995; Chen et al., 2014; Jouvenet et al., 2009; Kutluay et al., 2014; Muriaux et al., 2001), is a crucial determinant for the observed miRNA-mediated viral blockage effect.

Our new findings linking miRNAs and autophagy could have significant implications for retroviral therapeutics and for understanding how cells regulate retroviral assembly and potentially other RNA-mediated protein assembly processes. For example, miRNAs, provided they are not otherwise preoccupied with gene silencing, might be utilized therapeutically as a general inhibitor to disrupt or, if present in sufficient excess, even completely block formation of various retroviruses dependent on RNA-mediated Gag assembly. Additionally, the finding also raises the possibility of using small, nonsense synthetic nucleic acids as anti-retroviral agents against viral assembly. Furthermore, as the delivery and retention of endocytosed viral complexes in lysosomes is autophagy-dependent, this dependency could serve as an internal defense mechanism by cells to ensure that the virus does not exit the cells to infect other cells. Finally, as emerging evidence unravels the role of autophagy in mediating the clearance of RNA-protein aggregates (Frankel et al., 2017), the observed dependence on autophagy for degradation of misassembled viral complexes shown in this study could potentially serve as a model system for understanding how misassembled RNA-protein complexes are generally disposed of by cells. The new connection between miRNA-mediated disruption of viral assembly and autophagy could be crucial in broadening our understanding of the functions of miRNAs, providing new insights into how cellular behavior and disease evolution may be regulated when miRNAs function, rather than in their conventional role as gene silencers, as potent antagonists of RNA-scaffolded protein assembly processes.

## Materials and Methods

### Plasmid construction

HIV-1 proviral constructs including pNL43ΔPΔE, pNL43ΔPΔE-Gag-EGFP, pNL43ΔPΔEΔNC, and pNL43ΔPΔEΔNC-Gag-EGFP have been described previously (Chen et al., 2014). CMV-driven expression plasmids encoding HIV-1 Gag (pCR3.1-Gag) and HIV-1 Gag-mCherry (pCR3.1-Gag-mCherry) were kind gifts of Dr. Sanford Simons (Rockefeller University, New York, NY) and those that encode MLV Gag-EGFP (pcDNA3-MLV-Gag-EGFP) and SFV Gag-EGFP (pcDNA3-SFV-Gag-EGFP) were kind gifts of Dr. Stephan Gould (Johns Hopkins University, School of Medicine, Baltimore, MD). To generate pCR3.1-Gag-EGFP, the coding region of EGFP was first PCR amplified from pEGFP-N1 vector (Clontech) with forward primer 5′-attgcggccgcatggtgagcaagggcgagga-3′ and reverse primer 5′-atttctagattacttgtacagctcgtccatg-3′. The PCR product was then inserted into PCR3.1-Gag-mCherry backbone digested with *Not*I and *Xba*I to excise mCherry. To generate pcDNA3-MLV-Gag and pcDNA3-SFV-Gag, EGFP was first removed from pcDNA3-MLV-Gag-EGFP and pcDNA3-SFV-Gag-EGFP using *Bam*HI and *Xba*I, followed by end-filling the digested vector with Klenow and self-ligation of the resulting blunt-ended vectors. This operation leads to a stop codon in frame with the Gag sequences. To construct pcDNA3-MLV-ΔNC-Gag and pcDNA3-MLV-ΔNC-Gag-EGFP, plasmids that encode MLV Gag or MLV Gag-EGFP that harbor deletion of the nucleocapsid domain, respectively, the cDNA sequences corresponding to amino acids 16 to 23 in the NC domain of MLV Gag were deleted by PCR-mediated site-directed mutagenesis with forward primer 5′-GACCAGTGCGCCTACTGCAAGGAGAAG-3′ and reverse primer 5′-CTCGCCGCCCTGGCGGTCCTGCTTCTGGC-3′. shRNA constructs, including shRNA-ATG5 (TRC number: TRCN0000150645; clone ID: NM_004849.1-1043s1c1), shRNA-ULK1 (TRC number: TRCN0000000837; clone ID: NM_003565.x-535s1c1), and shRNA-controls were purchased from Sigma Aldrich.

### Antibodies and chemicals

Anti-LC3A/B rabbit polyclonal antibody was purchased from Cell Signaling Technology. Anti-p62 mouse monoclonal antibody and Alexa®Fluor 647-labeled Anti-LAMP1 mouse monoclonal antibody were purchased from Santa Cruz Biotechnology. Anti-ATPB mouse monoclonal antibody was purchased from ABCAM. Anti-LAMP3 (CD63) mouse monoclonal antibody was purchased from BD Pharmingen. Alexa®Fluor 594 donkey-anti-rabbit, Alexa®Fluor 594 goat-anti-mouse secondary antibodies, anti-GFP rabbit monoclonal antibody, and goat-anti-mouse secondary antibody HRP were purchased from Life Technologies. Anti-ATG5 mouse monoclonal antibody was purchased from MBL. Anti-GAPDH mouse monoclonal antibody was purchased from Sigma Aldrich. Pooled Ig from HIV-1-infected patients (HIV-Ig) was obtained from the NIH AIDS Research and Reference Reagent Program. Rapamycin and leupeptin were purchased from Sigma Aldrich. Bafilomycin A1 was purchased from Cell Signaling Technology. DAPI was purchased from Life Technologies.

### Cell culture

Wild type HEK 293 cells and HEK 293 cells overexpressing exogenous human hsa-miR-146a (MiR^+^) or hsa-miR-888 (MiR^+^888) were cultured in Dulbecco’s Modified Eagle’s Medium (DMEM, Mediatech), supplemented with 10% (*v*/*v*) FBS (PAN^TM^ Biotech), 1× GlutaMAX^TM^ (Thermo Fisher) at 37°C, 5% (*v*/*v*) CO_2_, and 90% relative humidity. Design of miRNA expression plasmids and generation of miRNA cell lines have been described previously (Chen et al., 2014). All experiments were performed with cells at passage numbers between 5 and 25.

### Transfection

Transfection was performed with FuGENE® 6 (Promega) as per manufacturer’s protocols when cells reached 50%–70% confluency. For fluorescence imaging studies where fluorescent proteins (FP) are used, cells were transfected with a mixture containing both FP-tagged and untagged viral constructs at a 1:3 ratio. For shRNA knockdown studies, shRNA constructs and viral constructs were transfected at 3:1 ratio.

### Virus particle purification, Western blotting and analysis of viral release efficiency

To collect viral particles, culture supernatant harvested at 48 h post-transfection of viral constructs was centrifuged at 1,000 ×*g* for 10 min and then filtered through a 0.45 μm filter to remove cell debris and large aggregates. Subsequently, 20 μL of Dynabeads®280 streptavidin (Life Technologies), precleaned twice with 1× PBS, was added into every 10 mL of the supernatant in order to assist the visualization of pellet after ultracentrifugation (100,000 ×*g* for 1 h). The viral particles and the beads were resuspended in lysis buffer (0.5% Triton X-100, 50 mmol/L pH = 7.5 Tris-HCl, 300 mmol/L NaCl) containing protease inhibitor cocktail (1:100). The lysates and the beads were further separated by microcentrifugation. To collect cell lysates after removing culture supernatants, the cells were washed once in cold 1× PBS, trypsinized, pelleted, and finally lysed in lysis buffer containing protease inhibitor cocktail (1:100). Gag from both supernatant and the cell lysates were analyzed by SDS-PAGE on 10% acrylamide gels and transferred to Immobilon^TM^-P membranes (Millipore). Immunoblotting was carried out with HIV-Ig or anti-GFP antibodies. Release efficiency was calculated (at 48 h post-transfection) as the ratio of supernatant Gag to total Gag, both determined by densitometry analysis of Western blot images using Fiji software.

### Electron microscopy (EM)

Cells were washed twice with 0.1 mol/L sodium cacodylate buffer (pH = 7.4) followed by fixation with 2.5% glutaraldehyde in the same buffer for 2 h, and then post-fixed with 1% OsO_4_ for 1 h at room temperature. After rinsing several times in cacodylate buffer and distilled water, the cells were incubated in 0.1% tannic acid (in cacodylate buffer) for 30 min, and stained in 1% uranyl acetate for 1 h. They were washed again in distilled water and dehydrated in a graded ethanol series and embedded in SPI-Pon 812 resin (SPI Supplies, PA, USA). Ultrathin (70 nm) sections were cut using an ultramicrotome (UC7, Leica Microsystem), and collected on copper grids with a single slot, stained with uranyl acetate and lead citrate. Then the sections were observed under a Tecnai G^2^ 20 TWIN electron microscope at 120 kV and photographed with an Eagle (4k×4k) digital camera (FEI, Oregon, USA).

### Fluorescence microscopy

All fluorescent microscopy experiments were performed on an Olympus IX 83 motorized inverted fluorescence microscope equipped with a 20× UCPlanFL N 0.7NA or a 100× UPlanSApo 1.4NA objective lens, back-illuminated EMCCD camera (Andor), Sutter excitation and emission filter wheels and an MT-20E excitation source (Olympus). Images were acquired using the Olympus MT20 filter set for DAPI, EGFP and TAMRA and a filter set (ET620/60x, ET700/75m, T660lpxr, Chroma) for Cy5. All images were acquired using CellSens Dimension software. Three-dimensional image stacks were acquired with 0.25 µm increments in the z-direction. Results were analyzed with Fiji (Schindelin et al., 2012) or AutoQuant deconvolution software (MediaCybernetics).

### Fluorescence *in situ* hybridization

Procedures for imaging fluorescence *in situ* hybridization (FISH) of hsa-miR-146a utilizing TSA Plus signal amplification (PerkinElmer) have been described before (Chen et al., 2014). FISH of unspliced HIV-1 viral RNA and GAPDH mRNA was performed as previously described, with modifications (Chen et al., 2014; Zhao et al., 2016). Specifically, cells were fixed in PBS solution containing 4% (*w*/*v*) paraformaldehyde for 20 min at room temperature, washed with 1× PBS, and permeabilized at 4°C in 70% (*v*/*v*) ethanol overnight. On the next day, the cells were washed thrice with wash buffer containing 2× SSC and 10% (*v*/*v*) formamide and then incubated in hybridization buffer (10% (*w*/*v*) dextran sulfate, 2× SSC, 10% (*v*/*v*) formamide) containing 250 nmol/L singly Quasar®570-labeled oligonucleotide probes against GAPDH mRNA (Cat# SMF-2026-1, Bioresearch) or 100 nmol/L singly TAMRA-labeled oligonucleotide probes against unspliced HIV-1 viral RNA (Chen et al., 2014) for 24 h at 37°C in a cell culture incubator. Prior to microscopy imaging, slides were washed thrice with wash buffer and then incubated in wash buffer for 30 min at 37°C, followed by two washes with 2× SSC and a final wash in 1× PBS to remove the unbound probe. Cells were incubated in 1× PBS for imaging.

### Data analysis

All experiments were repeated at least three times unless otherwise stated. Statistics were performed using Student’s *t*-test or one-way ANOVA with post-hoc testing of pairwise comparisons using Fischer’s Protected Least Significant Difference. Significant difference (indicated by *) was taken at the *P* < 0.05 level.

## Electronic supplementary material

Below is the link to the electronic supplementary material.

Supplementary material 1 (DOC 1221 kb)
