# Supplementary material for: Inhibition of retroviral Gag assembly by non-silencing miRNAs promotes autophagic viral degradation
Source: Protein Cell. 2017 Sep 7;9(7):640–51. doi: 10.1007/s13238-017-0461-z (PMC6019656; doi:10.1007/s13238-017-0461-z)

**Inhibition of retroviral Gag assembly by non-silencing miRNAs promotes autophagic viral degradation**

Na Qu^1^, Zhao Ma^1^, Mengrao Zhang^1^, Muaz N. Rushdi^1, 2^, Christopher J. Krueger^1, 2^ and Antony K. Chen^1,*^

**SUPPLEMENTARY FIGURE LEGENDS**

**Supplementary Figure 1. HIV-1 Gag forms large intracellular vacuoles in miR**^+^**888 cells.** The extent of Gag vacuole formation in miR^+^888 and Ctrl cells, both transfected with pNL43ΔPΔE-Gag-EGFP (see Materials and Methods), was monitored over time by fluorescence microscopy. Plotted is the time-course quantification of the percentage of cells with large intracellular Gag vacuoles. Data represent mean ± SD of three replicate experiments. Each replicate experiment was performed by examining at least 200 transfected cells.

**Supplementary Figure 2. The effect of co-expressing dynamin-K44A on Gag vacuole formation in miR**^+^**888 cells.** 48 h after co-transfection of pNL43ΔPΔE-Gag-EGFP alongside either a plasmid encoding Dyn-K44A or an empty control vector, percentage of cells with large intracellular Gag vacuoles was quantified. Data represent mean ± SD of three replicate experiments. Each replicate experiment was performed by examining at least 200 transfected cells.

**Supplementary Figure 3. Large intracellular vacuoles were not present in** M**iR**^+^ **cells not expressing Gag proteins.** MiR^+^ cells were fixed and immunostained with antibodies against LAMP3. Scale bar = 10 μm.

**Supplementary Figure 4. Overexpressing miRNA-146a does not affect LC3 level and conversion capacity**. a) MiR^+^ and Ctrl cell lysates were immunoblotted with antibodies against LC3 and GADPH as a loading control. b) Quantification of LC3 level, assayed by dividing total LC3 (LC3i plus LC3ii) by total GADPH. c) Quantification of LC3 conversion, assayed by dividing LC3ii by total GAPDH. Results are mean ± SD of at least three replicate experiments. Results were normalized to LC3 level or conversion capacity in Ctrl cells.

**Supplementary Figure 5.** **Localization of LC3 and HIV-1 Gag proteins in Ctrl cells.** 48 h after Ctrl cells were transfected with pNL43ΔPΔE-Gag-EGFP constructs, cells were fixed and immunostained with antibodies against LC3. LC3 and HIV-1 Gag both exhibit a dispersedly-distributed punctate staining pattern but do not colocalize. Scale bar = 10 μm.

**Supplementary Figure 6. The effect of knockdown of ULK1 on HIV-1 Gag vacuole formation.** shRNA-ULK1 (or shRNA-control) and pNL43ΔPΔE-Gag-EGFP were co-transfected in MiR^+^ cells. 48 h post-transfection, the extent of vacuole formation was assayed by fluorescence microscopy. Data represent mean ± SD of three replicate experiments. Each replicate experiment was performed by examining at least 200 transfected cells.

**Supplementary Figure 7. The effect of protease-inhibitor leupeptin on lysosomal sequestration of mitochondria and Gag.** 24 h after MiR^+^ cells were transfected with pNL43ΔPΔE-Gag-EGFP constructs, cells were treated with 0.25 mM leupeptin or DMSO control. Following 24 h incubation, cells were fixed and immunostained with antibodies against ATPB, a marker for mitochondrial inner membrane. a) Representative images of Gag and ATPB. Scale bar = 10μm. b) Gag enrichment in the vacuole lumen in response to leupeptin treatment. Plotted is the percentage of Gag vacuole-containing MiR^+^ cells with Gag enriched in the vacuole lumen, determined based on intensity measurements of a line scan across the vacuole. Data represent mean ± SD of at least three replicate experiments. Each replicate experiment was performed by examining at least 200 transfected cells.

**Supplementary Figure 8. The effects of overexpressing non-silencing miRNAs on VLP production of SFV Gag.** MiR^+^ and Ctrl cells were transfected with plasmids encoding CMV-driven SFV Gag. Western blot was performed and virus release efficiency was calculated as described in Materials and Methods without normalization. Data represent mean ± SD of at least three replicates.

**Supplementary Figure 9. The effects of overexpressing non-silencing miRNAs on intracellular localization of SFV Gag** a) Representative images of SFV Gag (detected by Gag-EGFP) in MiR^+^ cells. b) Percentage of cells with large intracellular Gag aggregates after transfection of SFV Gag. Data represent mean ± SD of three replicates. Each replicate experiment was performed by examining at least 200 transfected cells.

**Supplementary Figure 10. Effects of overexpressing hsa-miR-888 on the formation of intracellular vacuoles by HIV-1 and MLV Gag proteins.** MiR^+^888 cells or Ctrl cells were transfected with CMV-driven expression plasmids encoding HIV-1 or MLV Gag. At 48 h post-transfection, the percentage of cells having large intracellular vacuoles enriched in Gag was quantified. Data represent mean ± SD of at least three replicates. Each replicate experiment was performed by examining at least 200 transfected cells.

**Supplementary Figure 11. The effects of overexpressing hsa-miR-888 on intracellular localization of SFV Gag.** Percentage of cells with large intracellular Gag aggregates after transfection of SFV Gag. Data represent mean ± SD of three replicates. Each replicate experiment was performed by examining at least 200 transfected cells.

**Supplementary Figure 12. The effect of co-expressing dynamin-K44A on different Gag vacuole formation in MiR**^+^**888 cells.** MiR^+^888 cells were co-transfected with plasmids encoding HIV-1 or MLV Gag and Dyn-K44A or an empty vector. 48 h post-transfection, the percentage of cells having large intracellular vacuoles enriched in Gag was quantified. Data represent mean ± SD of at least three replicates. Each replicate experiment was performed by examining at least 200 transfected cells.

**Supplementary Figure 13. The effect of knockdown of ULK1 on MLV Gag vacuole formation.** shRNA-ULK1 (or shRNA-control) and CMV-driven plasmid constructs encoding MLV Gag were co-transfected in MiR^+^ cells. 48 h post-transfection, the extent of vacuole formation was assayed by fluorescence microscopy. Data represent mean ± SD of three replicate experiments. Each replicate experiment was performed by examining at least 200 transfected cells.

**Supplementary Figure 1.**

**
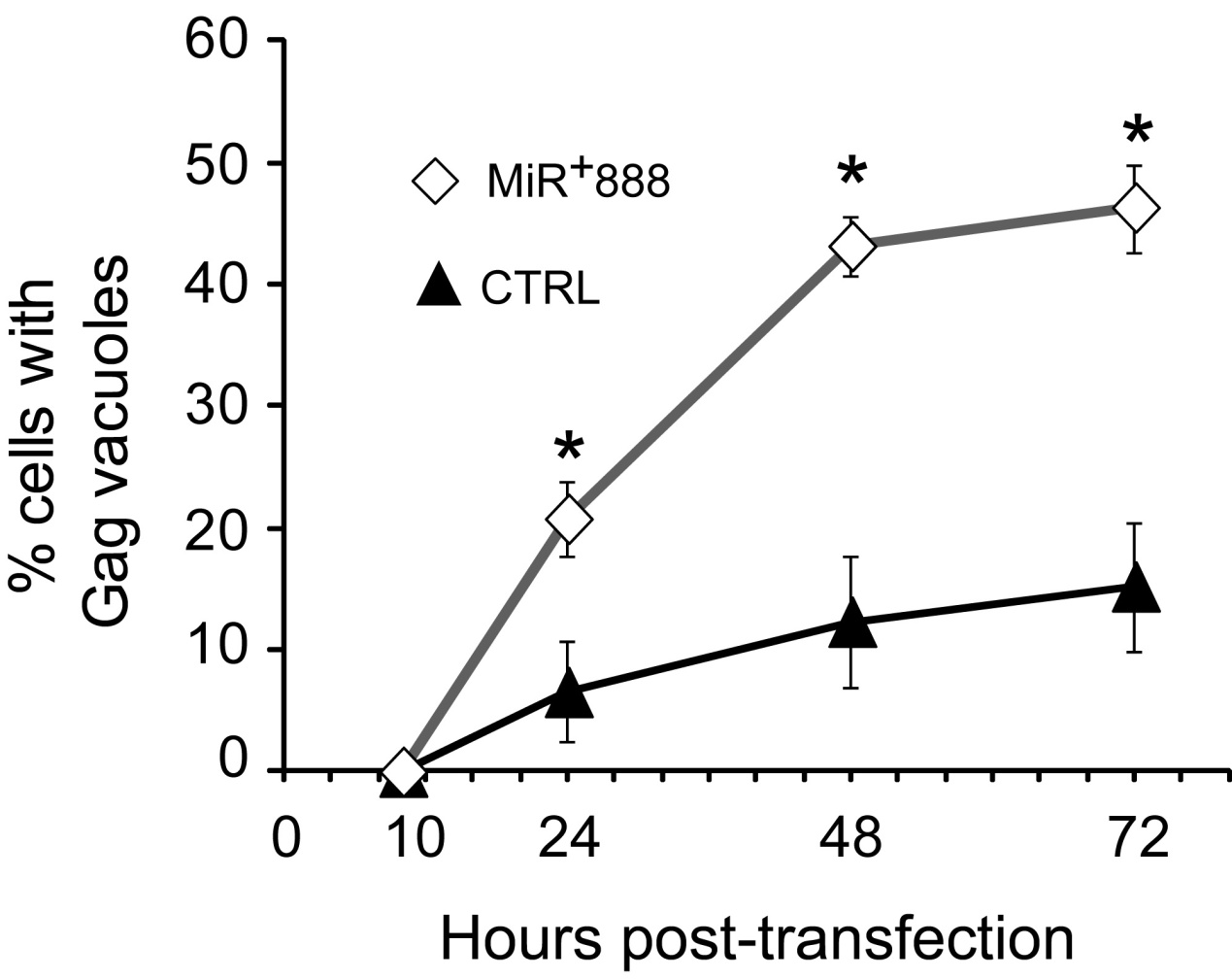
**

**Supplementary Figure 2.**

**
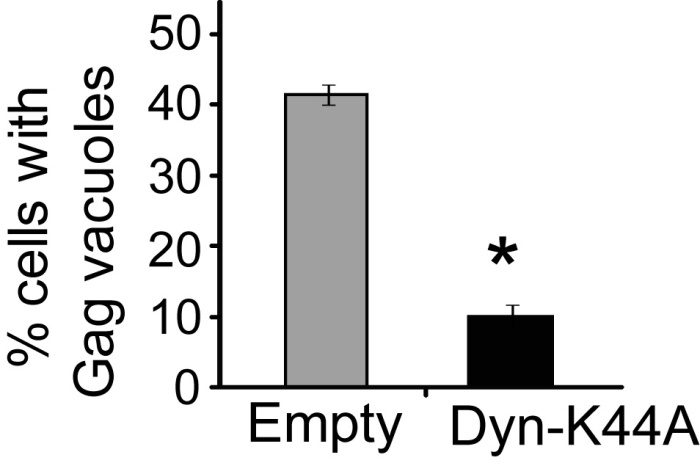
**

**Supplementary Figure 3.**

**
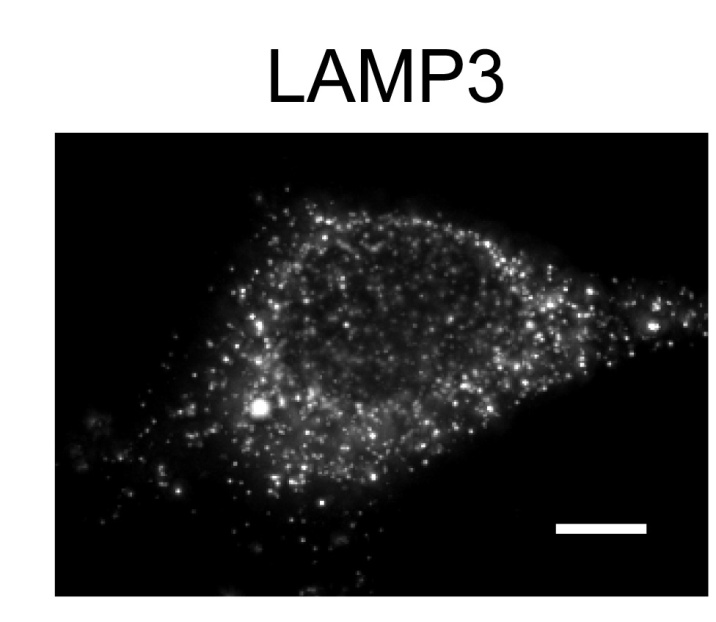
**

**Supplementary Figure 4.**

**
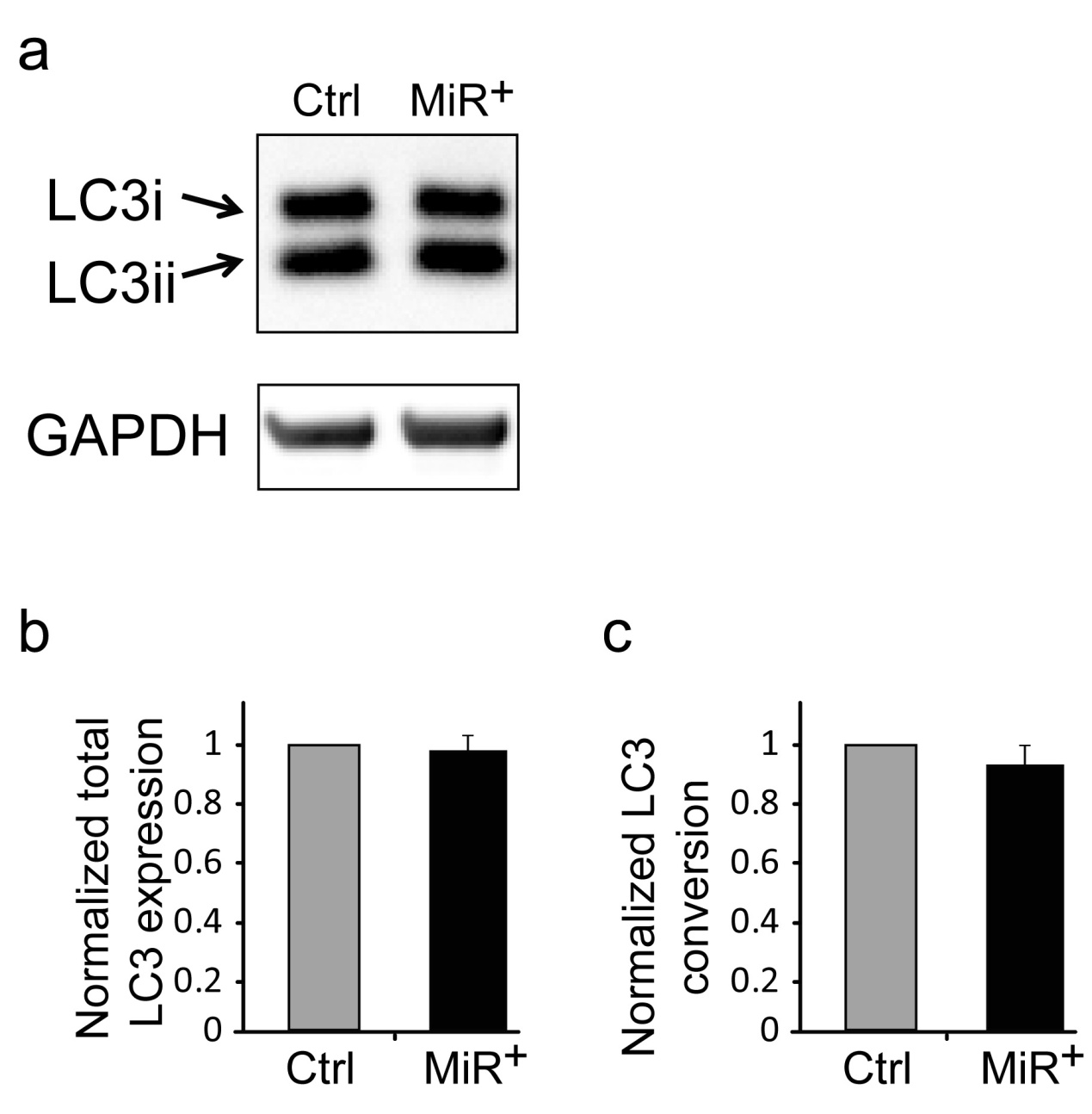
**

**Supplementary Figure 5.**

**
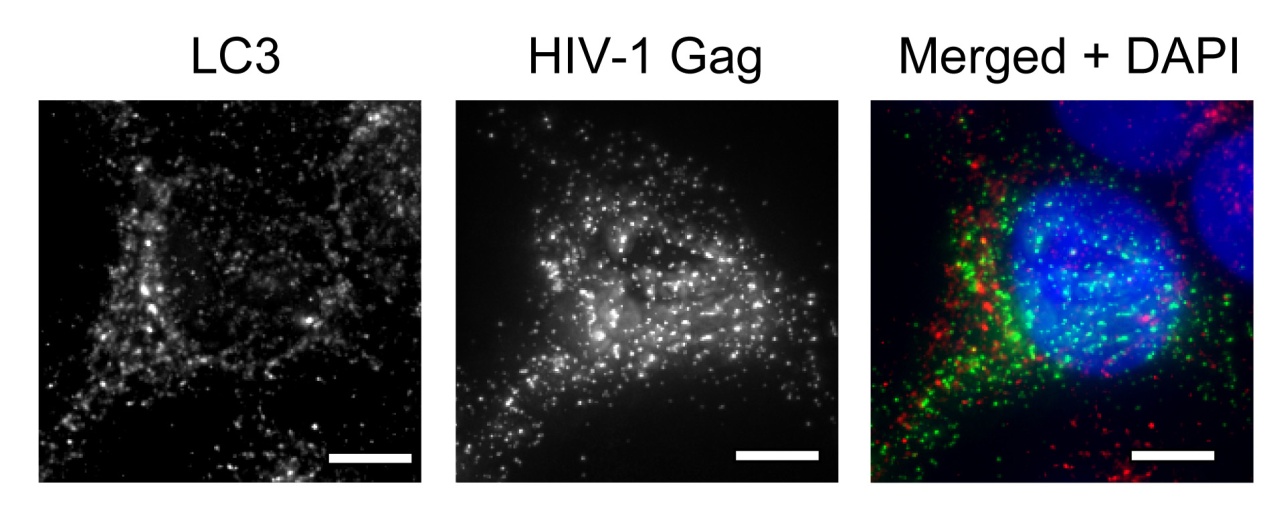
**

**Supplementary Figure 6.**

**
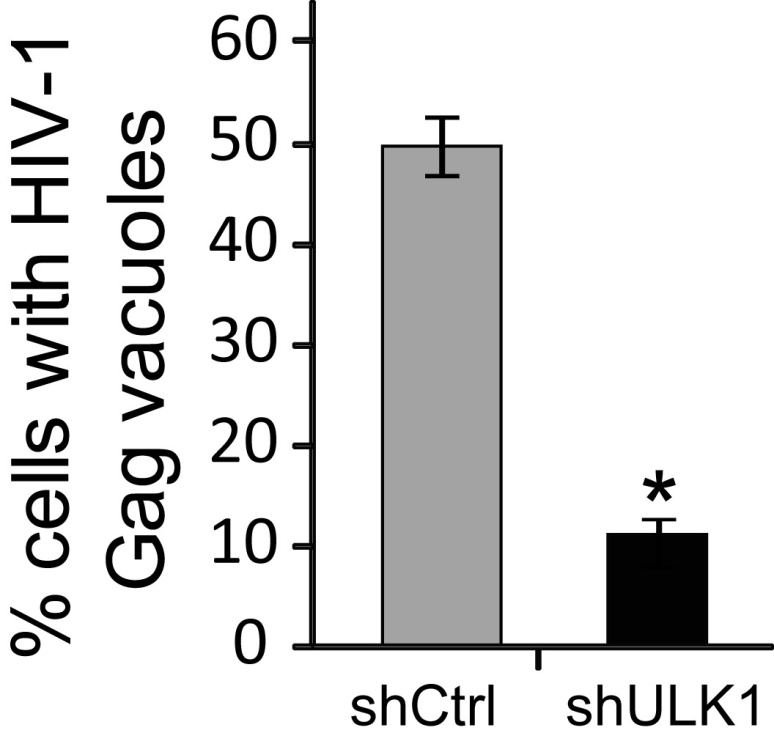
**

**Supplementary Figure 7.**

**­
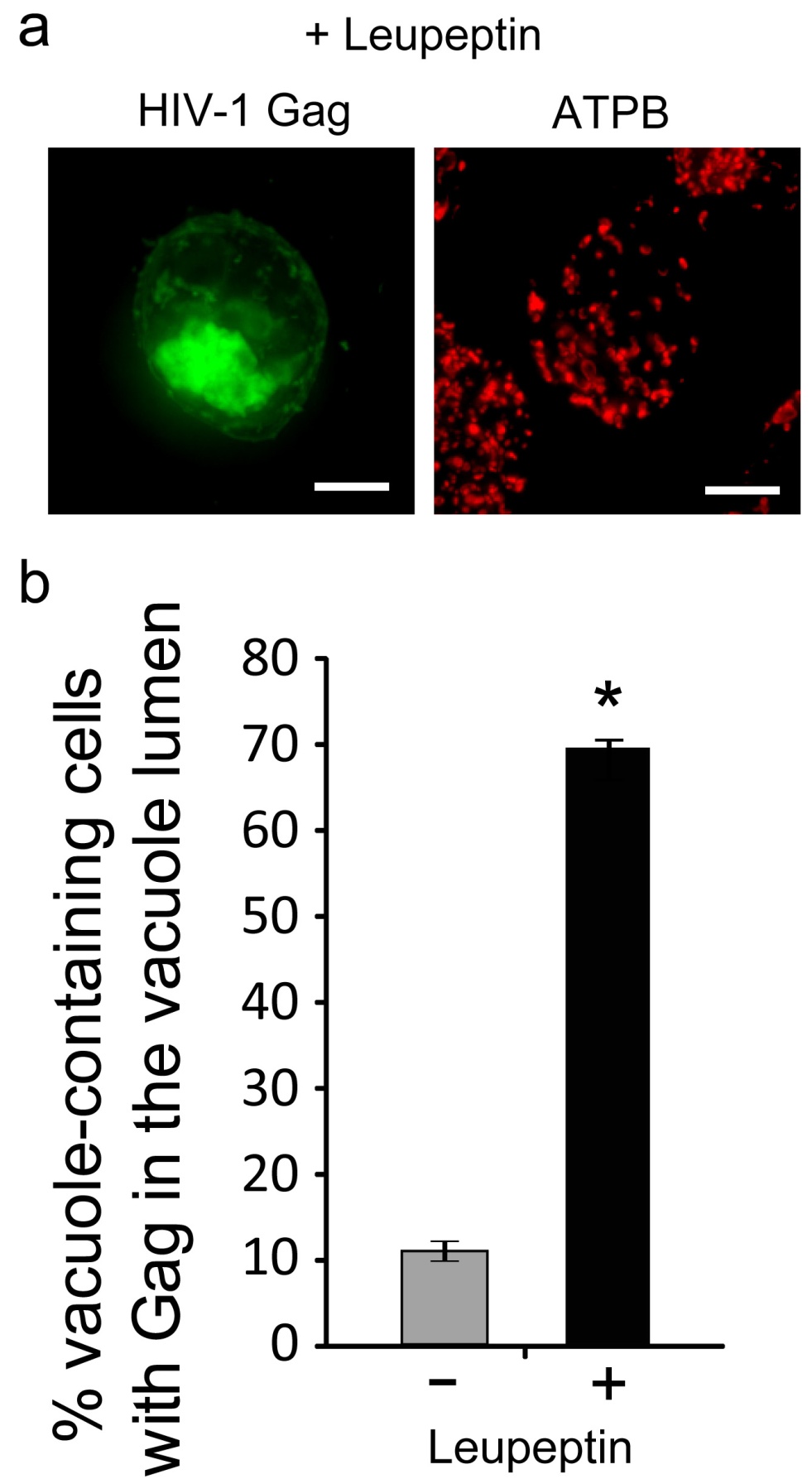
**

**Supplementary Figure 8.**

**
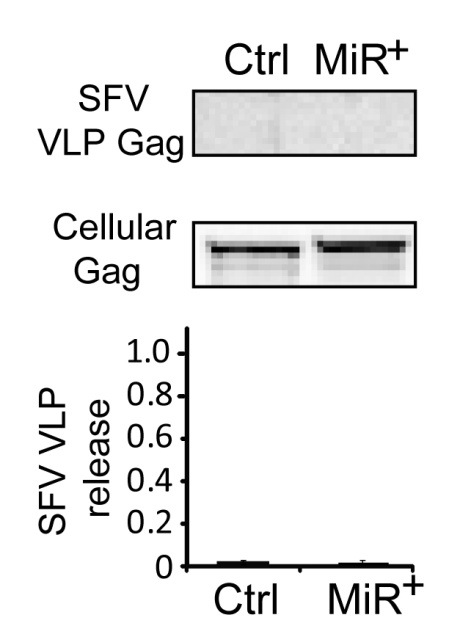
**

**Supplementary Figure 9.**

**
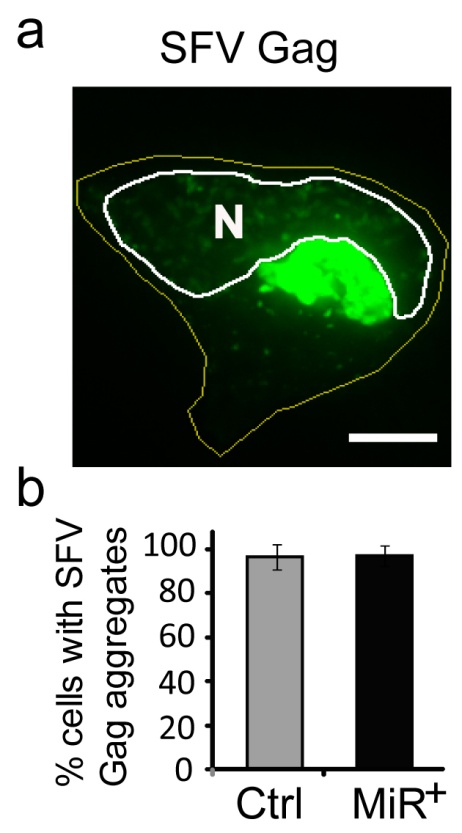
**

**Supplementary Figure 10**

**
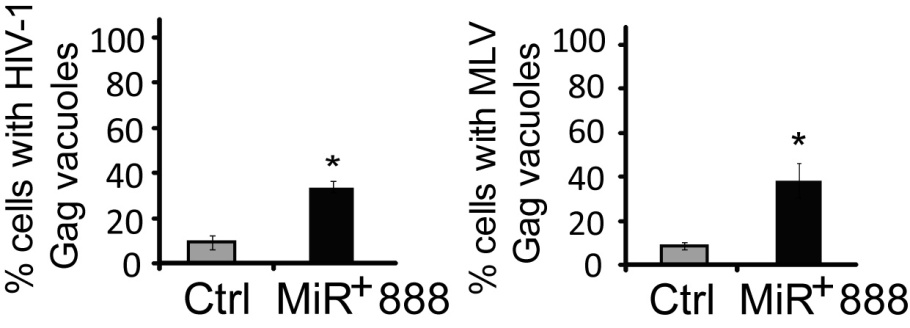
**

**Supplementary Figure 11**

**
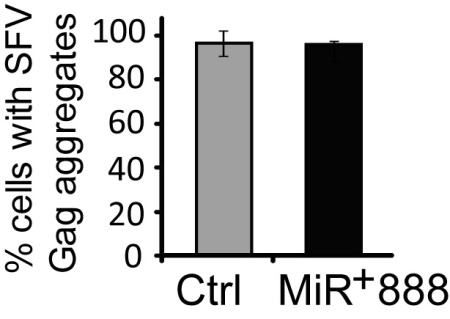
**

**Supplementary Figure 12.**

**
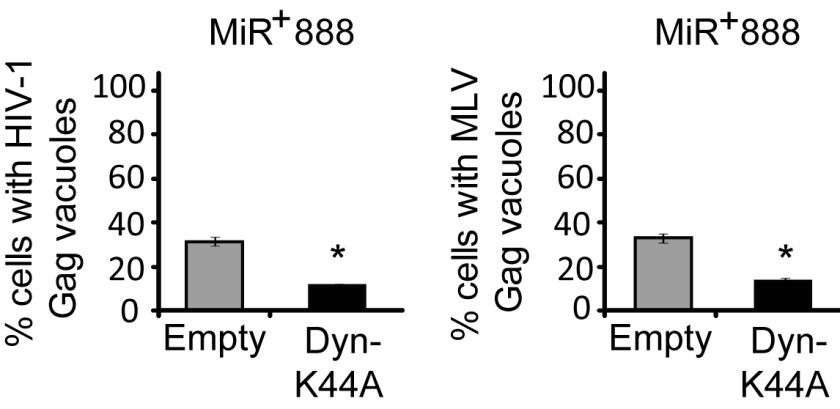
**

**Supplementary Figure 13.**


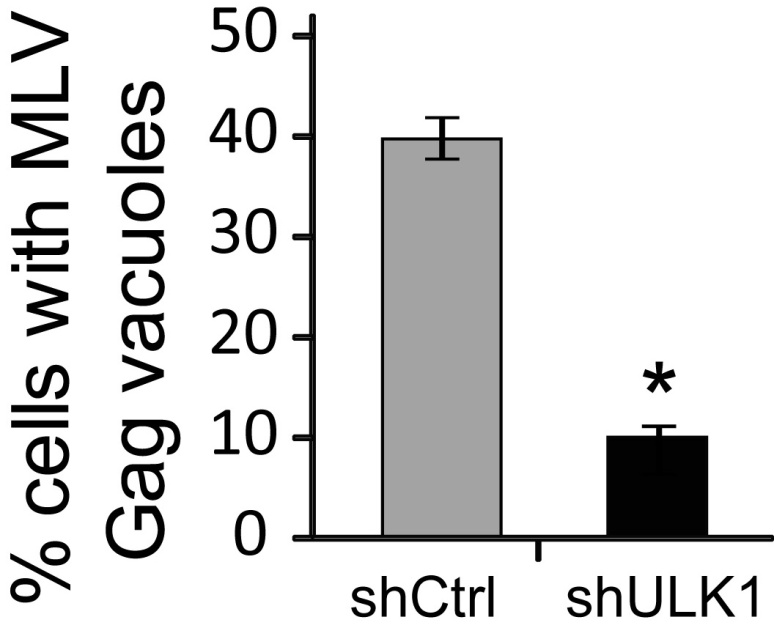

Supplement: Supplementary file 1 — Supplementary material 1 (DOC 1221 kb) [file 13238_2017_461_MOESM1_ESM.doc]
